# Synthesis of silver nanoparticles by sonogalvanic replacement on aluminium powder in sodium polyacrylate solutions

**DOI:** 10.1016/j.ultsonch.2022.105951

**Published:** 2022-02-16

**Authors:** Galyna Zozulya, Orest Kuntyi, Roman Mnykh, Andriy Kytsya, Liliya Bazylyak

**Affiliations:** a Department of Chemistry and Technology of Inorganic Substances, Lviv Polytechnic National University, 12 Bandery Str., 79013 Lviv, Ukraine; b Department of Physical Chemistry of Fossil Fuels of the Institute of Physical-Organic Chemistry and Coal Chemistry named after L. M. Lytvynenko of the National Academy of Sciences of Ukraine, 3a Naukova Str., 79060 Lviv, Ukraine

**Keywords:** Sonogalvanic replacement, Silver nanoparticles, Antimicrobial activity

## Abstract

The process of the formation of silver nanoparticles (AgNPs) via the method of galvanic replacement (GR) of Ag^+^ with aluminum powder in sodium polyacrylate (NaPA) solutions in the ultrasonic (US) field has been studied. It was observed, that the yellow colloidal solutions of stabilized AgNPs with the absorption maximum at ∼ 410 nm were obtained under the application of US power by 20 W and frequency by 20 kHz in the wide range of AgNO_3_ and NaPA concentrations (0.1 – 0.5 mM and 0.5 – 5.0 g/L respectively) at 25 ^0^C. It was shown, that the GR process under US field occurs without of the significant induction period. Using the UV–vis spectroscopy the kinetics of AgNPs formation has been studied and it was observed the first order kinetics with respect to Ag^+^ ions both for the nucleation and growth processes. It was found that observable rate constants of nucleation are close for the all experimental conditions but the observable rate constants of growth decreased with increasing of initial concentration of AgNO_3_. Based on the obtained kinetic data it was proposed a mechanism of the formation of AgNPs consisted of the following two main stages: 1) the nucleation with the formation of primary nanoclusters (AgNCs) on aluminum surface followed by their ablation from the surface of the sacrificial metal by ultrasound into bulk of solution; 2) the transformation of AgNCs in AgNPs via growth from the Al surface and / or agglomeration of AgNCs. Using TEM it was found that the size of obtained AgNPs does not exceed of 25 nm and slightly depends on the initial concentrations of precursors. High antimicrobial activity of obtained colloidal solutions against gram-negative and gram-positive bacteria as well as against fungi was observed.

## Highlights

• AgNPs synthesized via galvanic replacement by Al in surfactant solution at ultrasound.
• The kinetic peculiarities of the process were investigated.
• Antimicrobial activity of obtained solutions of AgNPs was studied.

## Graphical abstract

**Figure:**
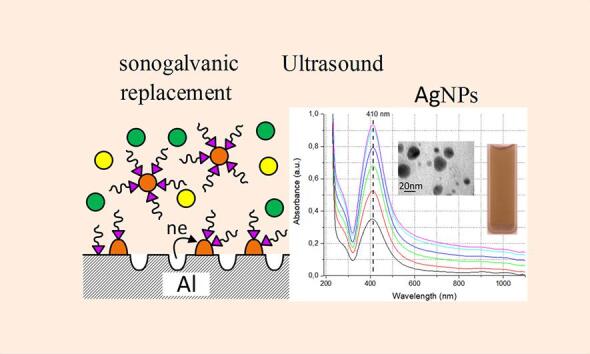

## 1 Introduction

**Fig. 1:**
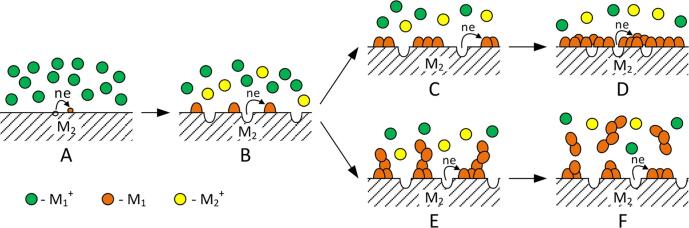
Scheme of galvanic replacement process with the formation of porous film coating (ABCD) or dendritic metal precipitate (ABEF) on the macrosurface of sacrificial substrate.

In the last decade, the galvanic replacement (GR) is often used to obtain the metal nanostructures [1], [2], [3], [4], [5] as well as for the synthesis of nanoparticles of metals (MNPs) [6], [7], [8]. It is worth noting, that the nanoparticles of sacrificial metal are used as the precursor. In *ref*. [9], [10], [11], [12], it has been shown that the macro-sized sacrificial metals can be used for the synthesis of MNPs under ultrasound assisted galvanic replacement reaction. Thus, the copper sheet [11] and the cobalt foil [10] are used for the synthesis of silver nanoparticles (AgNPs); the copper foil is used for the synthesis of gold nanoparticles (AuNPs) as well as the iron foil is used for the synthesis of platinum nanoparticles (PtNPs) [9]. Active sacrificial metals are used in order to synthesize the nanoparticles of non-noble metals applying the ultrasound assisted galvanic replacement reaction, in particular the aluminum foil for the CuNPs synthesis as well as the magnesium foil for the synthesis of iron nanoparticles (FeNPs), the cobalt nanoparticles (CoNPs) and tin nanoparticles (SnNPs) [10]. The formation of the precipitate from the reduced metal on the surface of sacrificial metals begins with the nucleation of nanoclusters (MNCs) during the galvanic replacement reactions. During the GR they grow with the formation of 1) a porous coating at high cathode polarization [13], [14], or 2) the dendritic coating at low cathodic polarization [15], [16], that is schematically illustrated on Fig. 1.

**Fig. 2:**
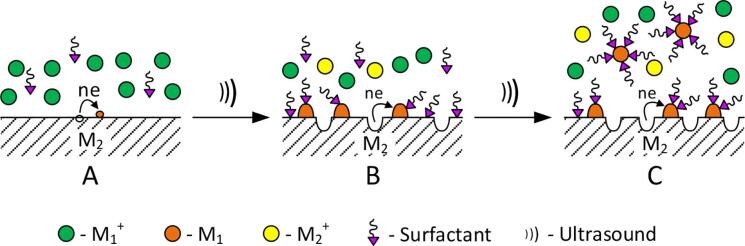
Scheme of the galvanic replacement process using the ultrasonic radiation on the macrosurface of the sacrificial substrate in the presence of surfactant.

In *ref.* [12], it has been shown that the substantial influence of the ultrasound is not observed only at the early stages of the galvanic replacement process, that is, during the formation of MNCs and MNPs on sacrificial surface. But in the subsequent nanoclusters and nanoparticles are separated and moved into a solution. So, the sacrificial surface is periodically updated, that provides a high and stable rate of the galvanic replacement reactions. Stabilized MNCs and MNPs (Fig. 2) are formed under the presence of the surfactant in solution. Surfactants at the expense of the adsorption and the formation of surface complexes with MNCs and MNPs cause the cathode polarization, which inhibits their growth and an agglomeration. Accordingly, the nanoparticles of small sizes are formed.

Galvanic replacement is a process that is occurred according to an electrochemical mechanism, during which the electrogenerating semi-reaction of the oxidation of sacrificial metal M_2_ on the anode regions (1) as well as the semi-reaction of the metal M_1_ reduction on the cathode regions (2) take place. Ultrasound causes an accelerating of the nucleation during the galvanic replacement, which contributes to the formation of a large number of MNPs with a uniform distribution of sizes in a short period of time, that is shown on example of AuNPs synthesis (∼10 nm) and PtNPs (∼6 nm) via the sonogalvanic replacement by copper and iron foils, respectively [9]. Thus, the ultrasound effect on the MNPs formation by GR is similar to the sonoelectrochemical synthesis of nanoparticles under using the sacrificial anodes [17], [18]. That is why, by analogy with sonoelectrochemical processes [19], a thinning of the electrode diffusion layer thickness takes place, which causes a significant increase in mass transfer (mass transport) near the electrode areas. As a result, the dissolution reaction of sacrificial metal (1) and the electrochemical reduction of the metal on the cathode regions (2), that is, the process of galvanic replacement (3), is accelerated. Finally, the ultrasound provides an ablation of nanoclusters and nanoparticles which are formed on the surface of sacrificial metal.

(1) $$\mathrm{on}\;anode\;area:\;nM_{2}\to nM_{2}^{m+}+me$$

(2) $$\mathrm{on}\;cathode\;area:mM_{1}^{n+}+ne\to mM_{1}$$

(3) $$mM_{1}^{n+}+nM_{2}↔mM_{1}+nM_{2}^{m+}$$

The sonogalvanic method is a relatively new direction in the synthesis of colloidal solutions of metal nanoparticles [20]. At the same time, it is practically not studied in terms of controllability of the MNPs formation. Therefore, the establishment of kinetic regularities of sonogalvanic synthesis of AgNPs in surfactant solutions on the example of sodium polyacrylate and sacrificial metal aluminum is the beginning of a systematic study of this method. The purpose of the work is to establish the regularities of “green” synthesis of antibacterial AgNPs synthesized via galvanic replacement process by aluminum powder using the ultrasonic radiation in solution of surfactant sodium polyacrylate. The value of the standard electrode potential of this sacrificial metal ($E_{Al^{3+}/Al}^{0}=-1.66V$) provides high electromotive force ($\Delta E=E_{M^{n+}/M}^{0}-E_{Al^{3+}/Al}^{0}$) and, accordingly, high process rate (3). Therefore, aluminum is promising as cheap and environmentally friendly sacrificial metal in hydrometallurgy of noble and nonferrous metals [21], [16]. In addition, GR by aluminum is used to obtain the nanostructured surfaces [22], [23]. $E_{M^{n+}/M}-E_{Al^{3+}/Al}$ Polyacrylate anion due to the O-donor atom forms the complexes with the ions of *d*-metals (Fe, Co, Ni, Cu, Zn, Cr) [24], [25], *s*-metals (Ca, Ba), *p*-metals (Al, Bi) and *f*-metals (Ce, Th, Nd) [25]. In *ref*. [26], [27], [28] the possibility of complex bond formation between the functional group COO − of polymer anion PA^–^ and the Ag^+^ ions is considered. PA^–^ anions also form the surface complexes with AgNPs with the formation of a peculiar nanostructure of Ag@PA, which provides an effective stabilization of nanoparticles [26], [27], [28], [29], [30], [31], [32]. Sodium polyacrylate is also a non-toxic and relatively cheap and widely available surfactant.

## 2 Experimental section

### 2.1 Materials

The following reagents were used for the synthesis of AgNPs solutions, namely the argentum nitrate AgNO_3_ (99.9 %, Alfa Aesar) was applied as a precursor of the metal, the sodium polyacrylate (NaPA) with MW = 2000 (45 % aqueous solutions, Sigma-Aldrich) was used as a surfactant, and the aluminum powder (99.5 %, Alfa Aesar, fraction 0.2…0.3 mm) was applied as the reducing agent.

### 2.2 Synthesis of silver Nanoparticles.

The research was carried out in the following directions: the first is the synthesis of solutions of silver nanoparticles stabilized by polyacrylate anion by galvanic replacement in the field of ultrasound; the second is the determination of their main characteristics, namely the absorption spectra, nanoparticle sizes and an establishment of antibacterial activity.

Synthesis of AgNPs performed in a solution of sodium polyacrylate and AgNO_3_, which was placed in a glass thermostated reactor connected to an ultrathermostate MLW UH8. The study was performed using an ultrasonic emitter magnetostrictive type Bandelin Sonopuls HD 2200.2 (Germany) at a power of 20 W and frequency of 20 kHz. The working element of the ultrasonic emitter was a titanium horn (diameter − 12 mm). The aluminum powder was added to working volume (50 mL) of sodium polyacrylate solution, turned on the ultrasound emitter adding a solution of AgNO_3_. Periodically (1, 2, 4, 6, 10, 15 and 20 min) solution samples were taken for the study of UV–vis spectra. After completion of the experiment, aluminum powder was separated from AgNP solution by decantation. The latter were stored in hermetically sealed glass containers made of dark glass.

The synthesis of AgNPs was performed at 25 °C and concentrations of AgNO_3_ 0.1…0.5 mM, NaPA 0.5…5 g⋅L^-1^; the quantity of aluminum powder was 0,25 g; pH = 9.0.

### 2.3 Characterization of AgNPs.

#### 2.3.1 UV–Vis spectroscopy

The samples of solutions were analyzed using the UV-3100PC UV–Vis-spectrophotometer (Shanghai Mapada Instruments Co., Ltd. (China)) in quarts cuvettes by 1 cm in thickness in the wavelength region from 190 to 1100 nm. The distilled water was used as the comparison solution.

#### 2.3.2 TEM analysis

TEM images of the samples were recorded using a JEM-I230 (JEOL, Tokyo, Japan) operating at an accelerating voltage of 80 kV. TEM grids were preliminary supplied a formvar film which then fixed by carbon using a JEE-4X vacuum evaporator (JEOL, Tokyo, Japan). Small drops (0.01–0.05 μL) of the silver compositions were applied to grids under the light microscope and were dried in air at room temperature. The sizes of obtained AgNPs were determined using TEM images by comparison of the sizes of individual particles with the scales presented on images.

#### 2.3.3 Antimicrobial activity of AgNPs

The antimicrobial activity AgNPs was evaluated against gram-negative bacteria *Escherichia Coli* ATCC 25,922 (*E. coli*) and gram positive bacteria *Staphylococcus aureus* ATCC 25,923 (*S. aureus*) and fungicidal activity was evaluated against diploid fungus *Candida albicans* ATCC 885–653 (*C. albicans*). To do this, the bacteria were inoculated into Petri dishes with a solid selective nutrient medium for each species of microorganisms: yolk-salt agar was used for *S. aureus,* Endo agar was used for *E. coli* and Sabouraud agar was used for *C. albicans*. Inoculation was performed after 1, 6, 18, 48 h of contact of bacteria with solution of AgNPs. The all of the biological materials were incubated at 310 K for 24 h in a bacteriological incubator. Antibacterial activity was indexed by counting of the number colony-forming units of microorganisms (CFU/mL). The method of colony counting was used to count the number of viable microorganisms.

## 3 Results and discussion

### 3.1 General features of the sonogalvanic replacement process

The presence of a dense oxide film on the surface of aluminum complicates the process of galvanic replacement despite the low value of the standard electrode potential of this sacrificial metal. By this is explained the much lower rate of reduction of silver by aluminum compared to copper [19], for which the standard electrode potential on ∼ 2 V ($E_{Cu^{2+}/Cu}^{0}$=0.34 V) is higher. In order to dissolve the film based on Al_2_O_3_ and to prevent its formation, the anions Cl^–^ [16] or F^–^ [22] are added into solutions, in which the galvanic replacement is carried out. However, in solutions containing the Ag(I) salts, the chloride–ions cause the formation of AgCl precipitate, and the fluoride–ions are undesirable for the AgNPs-based antibacterial drugs. Therefore, the alkaline solutions are alternative, in which the oxide film is dissolved via reaction (4). In addition, a semi-reaction (5) takes place in alkaline solutions during the galvanic replacement at the anode sections of the aluminum surface.

(4) Al_2_O_3_ + 2OH^–^ + 3H_2_O → 2[Al(OH)_4_]

(5) Al + 4OH^-^ → [Al(OH)_4_]^-^ + 3e, EAl(OH)4-/Al+4OH-0= −2.35 V

**Fig. 3:**
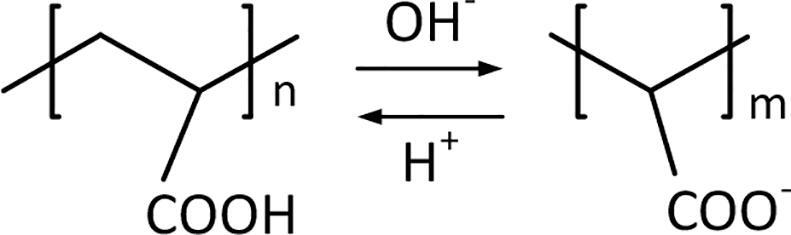
Scheme of the equilibrium process of transition of molecular (COOH) and ionic (COO^–^) forms in the polymer chain of polyacrylate depending on the acidity of the solution.

In solutions at pH = 6–11 in the polyacrylate polymer chain there are structural elements COOH and COO^–^(). The fraction of the latter is increased with increasing the concentration of OH^–^ (Fig. 3) and at pH ≥ 10 is in the anionic form only [33]. Ag^+^ ions are bonded with the anionic form (COO^–^) with the formation of soluble complexes [28], [29], which can be written as [Ag_m_PA]^(n–m)–^. With the use of the anionic form, the formation of the surface complexes with AgNPs is also carried out, i. e. the stabilization of the latter takes place. Therefore, pH ≈ 9 for working solutions provides an effective action of polyacrylate as a ligand and surfactant and the aluminum as a sacrificial substrate.

**Fig. 4:**
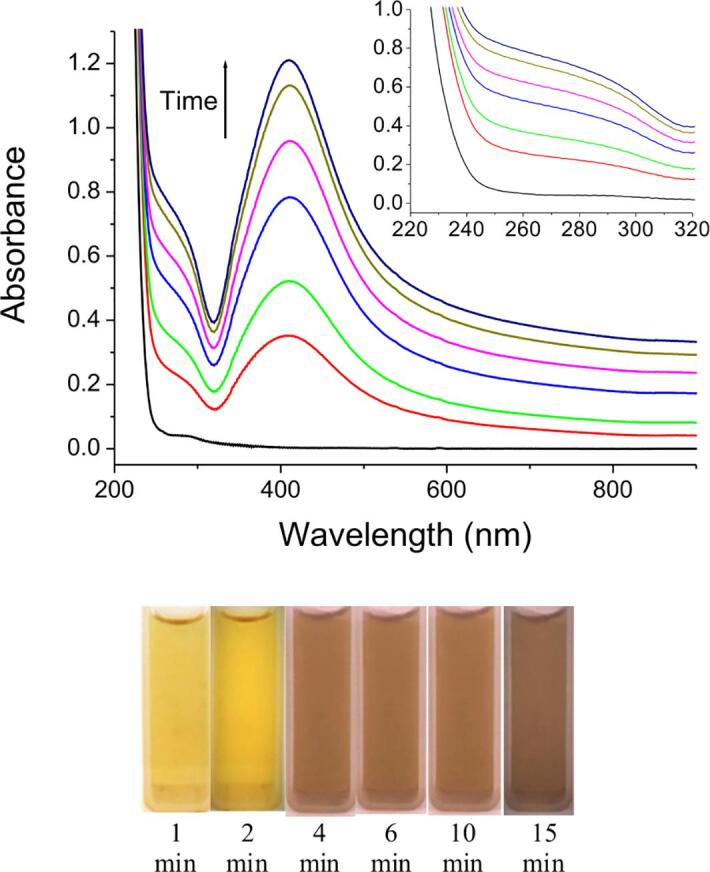
UV–Vis absorption spectra (*from above*) and images (*below*) of AgNPs solutions at different times of reaction. Insertion illustrates the section of UV–Vis spectra between 220 and 320 nm. Starting parameters of reaction mixture: C(NaPA) = 5 g⋅L^-1^; C(AgNO_3_) = 0.2 mM; temperature 25 °C.

The process of galvanic replacement of silver on the aluminum surface begins from the nucleation at the first seconds almost without an induction period [23], [34]. After that, the formed nanoclusters grow from the formation on the surface of the precipitate, the morphology of which depends on the conditions. In Ag^+^–PA^–^ solutions in a wide range of the concentrations of precursors at ultrasound, no silver precipitate is formed on the aluminum surface, i. e. galvanic replacement occurs according to the generalized equation (6) with the formation of colloidal solutions, as shown on Fig. 2. Yellow solutions with an absorption maximum at ∼ 410 nm, the value of which does not change during the sonogalvanic replacement process (Fig. 4) are obtained. The value of the maximum also does not shift during storage of solutions for a month, which indicates the stability of the synthesized AgNPs in NaPA solutions.

(6) [Ag_m_PA]^(n-m)-^ + m/3Al + 4 m/3OH^–^ → [Ag(0)_m_PA]^n-^ + m/3[Al(OH)_4_]

Ultrasound in aqueous solutions containing the organic substances (surfactants) causes the formation of radicals and new substances-precursors [35], in particular H^•^, R^•^, H_2_O_2_ (7), (8), (9), (10). The latter reduce the ions of silver (I) (11) with the subsequent formation of stabilized of AgNCs and AgNPs (12).

(7) H_2_O → H• + •OH

(8) RH + •OH(•H) → R• + H_2_O(H_2_)

(9) HO• + •OH → H_2_O_2_

(10) HO_2_• + •O_2_H → H_2_O_2_ + O_2_

(11) [Ag_m_PA]^(n-m)-^ + (H•, R•, H_2_O_2_) → [Ag(0)_m_PA]^n-^

(12) [Ag(0)_m_PA]^n-^ → AgNCs@PA → ANPs@PA

**Fig. 5:**
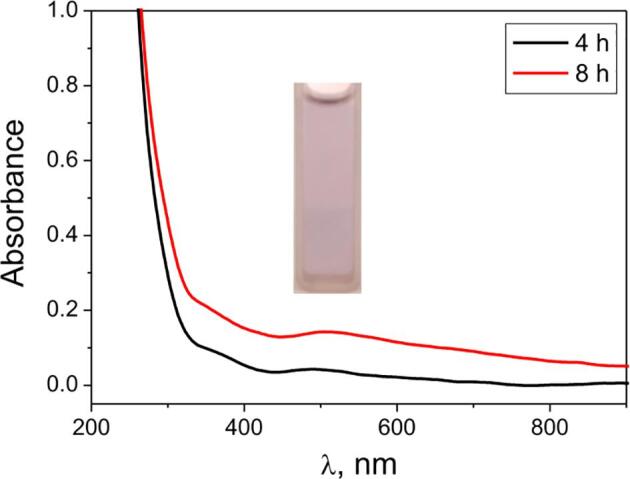
UV–Vis absorption spectrum and image of AgNPs solution after US treatment of solutions of 5 g⋅L^-1^ NaPA and 0.2 mM AgNO_3._

In the absence of aluminum in Ag^+^–PA^–^ solutions, a maximum at ∼ 520 nm of low intensity and weak color of the solution (Fig. 5) are observed under long-term action of ultrasound, which indicates a low content of nanoparticles. This confirms the priority of the sonogalvanic replacement by aluminum in the synthesis of AgNPs. Therefore, the main effect of ultrasound is to accelerate the process of galvanic substitution (6) and the ablation of AgNCs and AgNPs from the aluminum surface.

**Fig. 6:**
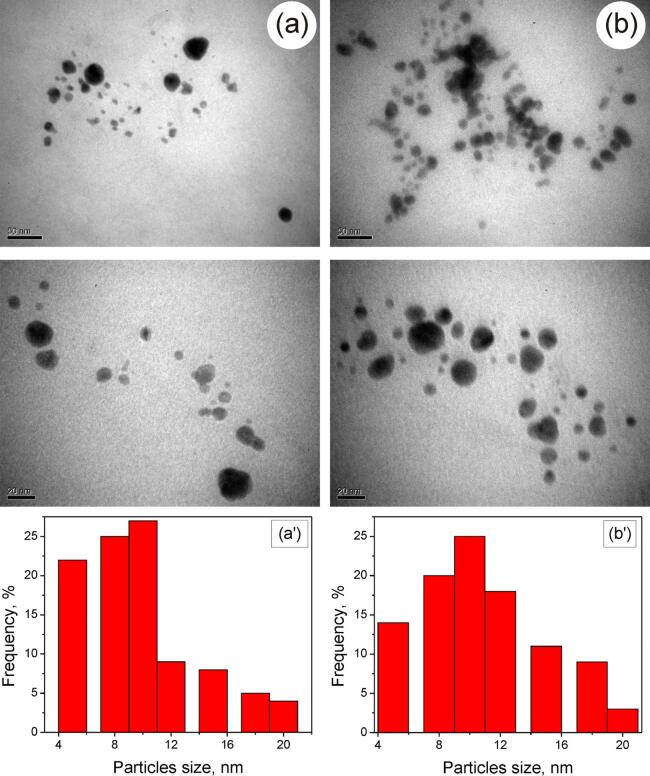
TEM images (*a, b*) and the size distribution histograms (*a', b'*) of AgNPs synthesized in solutions of 5 g⋅L^-1^ NaPA and 0.1 mM (*a, a'*) and 0.2 mM (*b*, *b'*) AgNO_3_ after sonogalvanic replacement by aluminum powder during 20 min.

The size of AgNPs synthesized by sonogalvanic replacement by aluminum in AgNO_3_–NaPA solutions does not exceed 25 nm (Fig. 6). The sizes of nanoparticles do not increase significantly with increasing the initial concentration of Ag(I) ions (Fig. 6, a′, b′), which explains the significant increase of their number (Fig. 6, a, b). The small sizes of AgNPs are due to the action of ultrasound, which accelerates the reduction of Ag(I) in the process of galvanic replacement (6) on the aluminum surface. The consequence of this is the predominance of the rate of nucleation over growth and, accordingly, the small size of AgNPs stabilized with polyacrylate as a surfactant.

### 3.2 An influence of the concentration of starting reagents

#### 3.2.1 An influence of the concentration of AgNO_3_

Recently it was reported that the optical characteristics of AgNPs solutions may be used for the evaluation of kinetics of their formation [36]. That is why in order to establish of kinetic peculiarities of the formation of AgNPs the spectra of the working solutions during the process of sonogalvanic replacement (Fig. 4) were used for the plotting of kinetic curves. As we can see on Fig. 4, the all spectra are characterized by well defined absorption band at ∼ 410 nm and by the shoulder at ∼ 290 nm. The absorption band at ∼ 410 nm is typical for AgNPs solutions and is caused by interaction of particles with the light (the phenomenon of surface plasmon resonance [37], [38]) and its intensity may be used for the evaluation of the rate of AgNPs growth. Besides, the shoulder at ∼ 290 nm may be attributed to the absorption of small (consisted of a few atoms of silver) so-called “magic clusters” [27] and its intensity may be used for the evaluation of the rate of AgNPs nucleation [32].

**Fig. 7:**
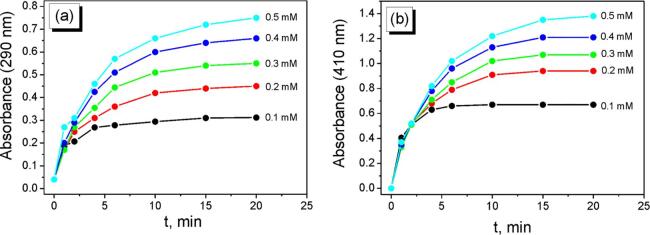
Dependencies of the change of optical density of solutions at 290 (a) and 410 (b) nm on time. The concentration of NaPA is 5 g/L.

The kinetic curves of AgNPs nucleation and growth for the different initial concentrations of AgNO_3_ are presented on Fig. 7. As it is clear from the Fig. 7, in the all studied cases the induction period is not observed which is usual for the processes of galvanic replacement of silver on the aluminum surface (vide supra). The analysis of obtained kinetic curves shown that both nucleation and growth of AgNPs may be fitted using first order reaction coordinates:

(13) $$\begin{array}{c} -\frac{d{\text{[Ag}}^{+}\text{]}}{\mathrm{dt}}=k\cdot {\text{[Ag}}^{+}\text{]}\\ \ln\left({\text{[Ag}}^{+}\text{]}\right)=\ln\left({\left[{\text{Ag}}^{+}\right]}_{\text{0}}\right)-k\cdot t \end{array}$$

In order to calculate the rate constants of nucleation and growth of AgNPs, the equation (13) was re-written as:

(14) $$\ln\left({\left[Ag^{+}\right]}_{0}\frac{A_{\mathrm{fin}}-A_{t}}{A_{\mathrm{fin}}}\right)=\ln\left({\left[Ag^{+}\right]}_{0}\right)-k\cdot t$$

Here *A_t_* is the absorbance at time *t* and *A_fin_* is the absorbance at the end of the process.

**Fig. 8:**
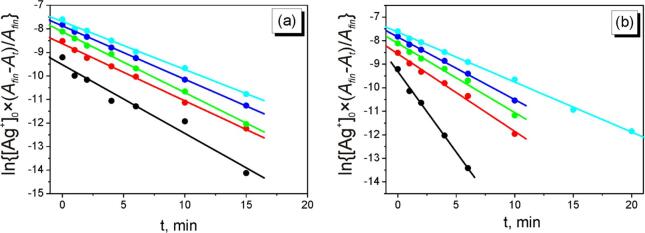
Kinetic curves of AgNPs nucleation (a) and growth (b) at different initial concentrations of AgNO_3_ in the coordinates of eq. (14).

As we can see on Fig. 8a, the all fitted straight lines are almost parallel and calculated rate constants of AgNPs nucleation (*k_N_*) are close for the initial concentrations of AgNO_3_ both 0.1 and 0.5 mM (Table 1). However, the rate constant of AgNPs growth for the initial concentration of AgNO_3_ 0.1 mM is higher in twice in comparison with the other experimental conditions.

**Table 1:** Calculated rate constants of the nucleation (*k_N_*) and growth (*k_G_*) of AgNPs at different initial concentrations of AgNO_3__._

[AgNO_3_]_0_, mM	*k_N_*, min^−1^	*k_G_*, min^−1^
0.1	0.29 ± 0.03	0.68 ± 0.02
0.2	0.24 ± 0.01	0.33 ± 0.02
0.3	0.26 ± 0.02	0.29 ± 0.02
0.4	0.23 ± 0.01	0.27 ± 0.01
0.5	0.21 ± 0.01	0.22 ± 0.01

This fact can be explained as follow. As we can see from TEM-images (Fig. 6) obtained AgNPs are quite polydisperse which indicates the agglomeration of particles at the stage of their growth. The formation of AgNPs in studied conditions occurs by gross-reaction (6) via the following sequence of physical–chemical transformations.

Adsorption of NaPA on the Al surface

(15) PA^n–^ + Al → [Al–PA]^n–^

After addition of Ag^+^ in the reaction mixture NaPA–Ag complexes are formed both in the solution ([Ag(I)_m_PA]^(n-m)-^) and on the Al surface (Al–[Ag(I)_m_PA]^(n-m)-^):

(16) [Al–PA]^n–^ + mAg(I) → Al–[Ag(I)_m_PA]^(n-m)-^

Exactly these absorbed complexes react with Al surface forming “magic clusters” (vide supra):

(17) Al–[Ag(I)_m_PA]^(n-m)-^ → Al–[Ag(I)_(m-x)_Ag(0)_x_PA]^(n-m+x)-^

Here the charge of product of reaction is unknown due to possibility of formation of polyatomic charged clusters such as Ag_2_^+^, Ag_4_^2+^ etc [27] as well as due to possible incomplete reduction of Ag(I). As it was mentioned above, in the absence of Al in reaction mixture the rate of AgNPs formation is much lower than in a case of sonogalvanic replacement process (see Fig. 4, Fig. 5). Therefore, the reactions (7), (8), (9), (10), (11), (12) can be neglected and the role of ultrasound is reduced to ablation of formed particles from the surface. Thus, formed “magic clusters” are shaking off from the Al surface and the shoulder at ∼ 290 nm is observed on the spectra of working solutions:

(18) Al–[Ag(I)_(m-x)_Ag(0)_x_PA]^(n-m+x)-^ → [Ag(I)_(m-x)_Ag(0)_x_PA]^(n-m+x)-^ +Al

As a result of shaking off the “magic clusters”, the aluminum surface is released and can adsorb new complexes with their subsequent reduction:

(19) Al + [Ag(I)_m_PA]^(n-m)-^ → Al–[Ag(I)_(m-x)_Ag(0)_x_PA]^(n-m+x)-^ → [Ag(I)_(m-x)_Ag(0)_x_PA]^(n-m+x)-^ +Al

Therefore the rate constants *k_N_* for the all of cases are close (Table 1).

At the same time, Al surface can adsorb not only complexes [Ag(I)_m_PA]^(n-m)-^ but also “magic clusters” leading to the full reduction of complexed Ag(I) and formation of primary nanoclusters (AgNCs):

(20) Al–[Ag(I)_(m-x)_Ag(0)_x_PA]^(n-m+x)-^ → [Ag(0)_m_PA]^n-^ +Al

The transformation of AgNCs in AgNPs (growth process) may occur by different routes:

1) due to the reduction of silver ions, free COO^–^ vacancies are released for the addition of new ions from the solution subsequent by reduction of formed complexes on Al surface:

(21) [Ag(0)_m_PA]^n-^ + xAg^+^ → [Ag(0)_m_Ag(I)_x_PA]^(n-x)–^ → [Ag(0)_(m+x)_PA]^n-^

2) agglomeration of AgNCs:

(22) x[Ag(0)_m_PA]^n-^ → [Ag(0)_(m×x)_PA]^n-^

Taking into account the above mentioned considerations we can assume the following statements. The rate constants of AgNPs nucleation not depend on the initial concentrations of AgNO_3_. Thus, the concentration of nucleus (AgNCs) is increased with the increase of Ag^+^ concentration. Increasing of concentration of AgNCs leads to an increase of the probability of their agglomeration and, respectively, to the decrease of the fraction of small (∼5 nm) particles compared to the fraction of bigger ones (8–15 nm) (see Fig. 6a' and 6b'). The agglomeration process leads to the decrease of the number of particles in the solution. Assuming that absorbance depends on both the number and the size of AgNPs [37], such effect leads to decreasing of the observable rate constant of AgNPs growth. That is why we can assume that the size of formed AgNPs is slightly increased with the increase of the initial concentration of Ag^+^ due to increasing of probability of agglomeration processes.

#### 3.2.2 An influence of the concentration of NaPA

An influence of the concentration of NaPA (C(NaPA)) on the kinetics of AgNPs formation at the initial concentration of AgNO_3_ 0.2 mM was studied in the wide range of C(NaPA) (0.5 – 5 g/L).

**Fig. 9:**
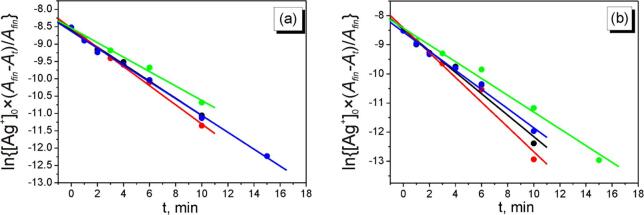
Kinetic curves of AgNPs nucleation (a) and growth (b) at different initial concentrations of NaPA in the coordinates of eq. (14).

It was found that the rate constants of nucleation and growth does not depend on the C(NaPA) in the studied concentration diapason (Fig. 9, Table 2). This fact can be explained as follow.

**Table 2:** Calculated rate constants of the nucleation (*k_N_*) and growth (*k_G_*) of AgNPs at different initial concentrations of NaPA.

C(NaPA), g L^–1^	*k_N_*, min^−1^	*k_G_*, min^−1^
0.5	0.25 ± 0.01	0.37 ± 0.04
1	0.28 ± 0.01	0.42 ± 0.05
2	0.21 ± 0.02	0.29 ± 0.05
5	0.24 ± 0.01	0.33 ± 0.02

It is well known that the activation energy of the process of galvanic replacement is quite low (8 – 14 kJ/mol [39]) confirming that the reaction is controlled by the mass transfer of the ions onto the surface of sacrificial metal. In our case the concentration of COO^–^ groups of NaPA is much higher than concentration of Ag^+^ (7 – 70 mM for COO^–^ and 0.2 mM for Ag^+^). That is means the all Ag^+^ ions are complexed by PA^–^ but the solution also contains the uncomplexed with silver macromolecules of polyacrylate. As it was mentioned above, the nucleation (or formation of “magic clusters”) is occurred via the reduction of absorbed complexes Al–[Ag(I)_m_PA]^(n-m)-^ on the Al surface (eq. (17)) and taking into account the high concentration of NaPA (more than 30 × excess compared to Ag^+^) the concentration of such absorbed complexes (and formed [Ag(I)_(m-x)_Ag(0)_x_PA]^(n-m+x)-^ both on the Al surface and in the bulk of solution) depends only on the concentration of ions of silver. Respectively, the rate of AgNPs growth depends on the concentration of these complexes (eqs. (20), (21)) and is not changed with the changing of NaPA concentration.

### 3.3 The antimicrobial activity of AgNPs

**Fig. 10:**
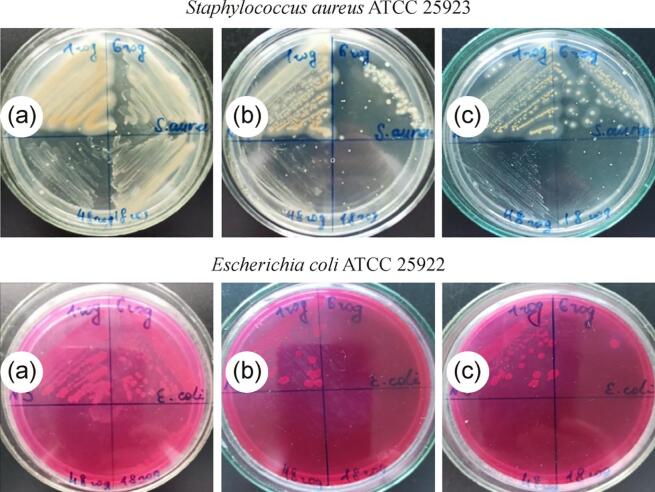
Photos of Petri dishes, illustrated the bactericidal effect of AgNPs solutions synthesized by sonogalvanic replacement method at initial concentrations of AgNO_3_ equal to 0.1 mM (*a*), 0.2 mM (*b*) and 0.5 mM (*c*).

**Fig. 11:**
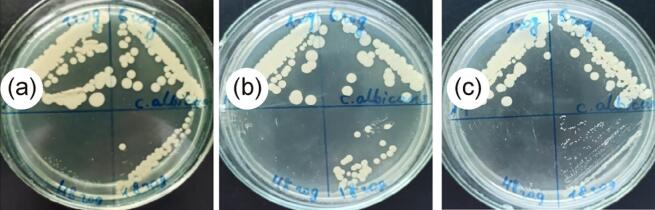
Photos of Petri dishes, illustrated the fungicidal effect of AgNPs solutions synthesized by sonogalvanic replacement method at initial concentrations of AgNO_3_ equal to 0.1 mM (*a*), 0.2 mM (*b*) and 0.5 mM (*c*) against *Candida albicans* ATCC 885–653.

The investigations of antimicrobial activity of synthesized AgNPs via sonogalvanic replacement method indicates on their efficiency against gram-positive bacteria *Staphylococcus aureus* ATCC 25,923 and gram-negative bacteria *Escherichia coli* ATCC 25,922 (Table 3, Fig. 10) as well as on their fungicidal activity against diploid fungus Candida albicans ATCC 885–653 (Table 4, Fig. 11). The highest antibacterial activity of AgNPs was observed against *E. Coli* which is a representative of gram-negative bacteria of the family of *Enterobacteriaceae* (Table 3, Fig. 10). Like of the most gram-negative bacteria *E. coli* is unable to form the spores and this fact may be one of the explanations of the high antibacterial activity of the synthesized silver nanoparticles against this bacterium. With increasing of initial concentration of AgNO_3_ from 0.1 to 0.5 mM the number of viable bacteria decreases by 80 % at an exposure time of 1 h. Complete cessation of bacterial growth occurs after contact with AgNPs solution for 48 and 6 h for the AgNPs solutions obtained at initial concentrations of AgNO_3_ equal to 0.1 and 0.2 mM respectively.

**Table 3:** Antibacterial properties of AgNPs solutions synthesized by sonogalvanic replacement method and stabilized with 5 g/L NaPA solution.

Species of microorganisms	Exposure time, h	Quantity colony-forming units /mL		
		0.1 mM AgNO_3_	0.2 mM AgNO_3_	0.5 mM AgNO_3_
*S. aureus* ATCC 25923 (F-49)	1	150	100	90
	6	90	40	20
	18	50	not found	not found
	48	20	not found	not found
*E. coli* ATCC 25922 (F-50)	1	100	30	20
	6	70	not found	not found
	18	25	not found	not found
	48	not found	not found	not found

**Table 4:** Fungicidal properties of AgNPs solutions synthesized by sonogalvanic replacement method and stabilized with 5 g/L NaPA solution.

Species of microorganisms	Exposure time, h	Quantity colony-forming units /cm^3^		
		0.1 mM AgNO_3_	0.2 mM AgNO_3_	0.5 mM AgNO_3_
*Candida albicans* ATCC 885–653	1	70	60	45
	6	50	30	20
	18	30	20	not found
	48	20	not found	not found

*S. Aureus* is a representative of the gram-positive microflora of the family of *Staphylococcaceae*. The antimicrobial activity of AgNPs synthesized by sonogalvanic replacement method against these bacteria is some lower (Table 3, Fig. 10). Increasing of the exposure time up to 6 h reduces the number of viable bacteria by 1.7 times for the AgNPs solution obtained at initial concentration of AgNO_3_ equal to 0.1 mM. With a further increase in the contact time by 3 and 8 times the amount of CFU decreases by 1.8 and 2.5 times respectively, which is corresponds to 86.7% of their number at the exposure of 1 h. In contrast to *E. coli* the complete cessation of the growth of bacteria using of AgNPs synthesized with 0.1 mM of AgNO_3_ is not observed. As it was expected, th increasing of the initial concentration of AgNO_3_ by 2 and 5 times naturally reduces the number of bacteria by 30 and 40% respectively and complete inactivation of *S. aureus* observed at an exposure time of 18 h.

The fungicidal activity of the synthesized via sonogalvanic replacement method AgNPs was studied on the example of the diploid fungus Candida albicans from the family of Saccharomycetaceae (Table 4, Fig. 11). Using AgNPs synthesized from 0.1 mM AgNO_3_, a decrease of the number of colony-forming units up to 20 was observed at 48 h of exposure, which is 71.4% compared to 1 h exposure. Increasing of the concentration of AgNO_3_ by 2 and 5 times reduces the time required to stop the growth of fungi to 48 and 18 h respectively.

The antimicrobial action of AgNPs synthesized by sonogalvanic replacement can be explained by the following mechanisms: 1) fixation of AgNPs on the cell membranes with their subsequent penetration into the cell, damage of the membrane and the release of cellular contents (so-called “Trojan horse” mechanism) [40]; 2) release of Ag^+^ ions which are characterized by antimicrobial properties.

The comparison of antimicrobial activity of NaPA-stabilized AgNPs synthesized by sonogalvanic replacement method and sonoelectrochemical method [17] indicates the absence of significant differences of their bactericidal effect against gram-negative bacteria *Escherichia coli* as well as against fungus *Candida albicans*.

## 4 Conclusions

The colloid solutions of AgNPs with an absorption maximum of ∼ 410 nm have been obtained using the galvanic replacement of silver ions with aluminum powder in the ultrasonic field in polyacrylate solutions. The formation of AgNPs occurs without of the significant induction period. The formation of AgNPs stabilized by NaPA via sonogalvanic replacement can be considered as a two-stage process, namely 1) the nucleation with the formation of small silver nanoclusters on the aluminum followed by their ablation from the surface of the sacrificial metal by ultrasonic field into solution and 2) the growth of AgNPs via the reduction of Ag(I)–PA complexes on Al surface or/and coagulation of AgNCs. Both the nucleation and the growth of AgNPs may be fitted using the first order kinetics equation with respect to Ag(I) concentration. In the wide range of initial concentrations of AgNO_3_ and NaPA the observable rate constants of nucleation are close and are within 0.21 – 0.29 min^−1^. At the same time the decrease of *k_G_* with increasing of initial concentration of AgNO_3_ was observed. The size of AgNPs synthesized via GR by aluminum in US in NaPA solutions does not exceed 25 nm and slightly dependent on the initial concentrations of precursors. The synthesized AgNPs demonstrated an efficient antibacterial activity against *Escherichia Coli* and *Staphylococcus aureus*, as well as fungicidal activity against *Candida albicans*. The most effective antibacterial effect of AgNPs was observed against *E. Coli*.

## CRediT authorship contribution statement

**Galyna Zozulya:** Investigation, Conceptualization, Writing – original draft. **Orest Kuntyi:** Conceptualization, Project administration, Writing – original draft, Writing – review & editing. **Roman Mnykh:** Methodology, Investigation. **Andriy Kytsya:** Data curation, Formal analysis, Writing – review & editing. **Liliya Bazylyak:** Formal analysis, Writing – original draft.

## Declaration of Competing Interest

The authors declare that they have no known competing financial interests or personal relationships that could have appeared to influence the work reported in this paper.
